# Ferroptosis: a new dawn in the treatment of acute lymphoblastic leukemia

**DOI:** 10.3389/fonc.2025.1669432

**Published:** 2026-01-09

**Authors:** Jiongping Han, Yifan Yang, Yuxin Wei, Jing Jin, Weiying Feng

**Affiliations:** Department of Hematology, Shaoxing People’s Hospital, The First Hospital of Shaoxing University, Shaoxing, China

**Keywords:** ferroptosis, acute lymphoblastic leukemia, molecular mechanism, ferroptosis-inducing drugs, ferroptosis-related genes

## Abstract

Acute lymphoblastic leukemia poses substantial challenges in therapeutic management. As a novel form of programmed cell death, ferroptosis has emerged as a promising research direction in cancer therapy. Studies have emphasized the pivotal role of ferroptosis in ALL, indicating that targeting this pathway could provide a novel therapeutic strategy. This article systematically reviews the molecular regulatory mechanisms of ferroptosis in ALL and explores its potential clinical applications in the treatment and prognostic evaluation of the disease.

## 1 Introduction

Acute lymphoblastic leukemia (ALL) is a malignant clonal disease originating from lymphocytes, and is classified into acute T-cell lymphoblastic leukemia (T-cell ALL, T-ALL) and acute B-cell lymphoblastic leukemia (B-cell ALL, B-ALL) (1). According to the latest report from the American Cancer Society, approximately 6,100 new cases of ALL are expected to be diagnosed in 2025, with an estimated 1,400 deaths (2). Current treatment regimens primarily involve multi-drug combination chemotherapy, consolidation and maintenance therapy, and bridging to allogeneic hematopoietic stem cell transplantation (3, 4). However, with prolonged chemotherapy, patients often develop drug resistance, cumulative toxicity, and bone marrow suppression, leading to an increased risk of relapse and mortality among ALL patients (5). For relapsed/refractory ALL, effective salvage therapies remain limited, underscoring the urgent need for novel treatment strategies. To improve the long-term prognosis and quality of life for ALL patients, it is essential to explore novel therapeutic approaches that are both highly effective and minimally toxic.

Since Dixon et al. first introduced the concept of “ferroptosis” in 2012, its unique mechanistic properties have garnered significant attention, opening new avenues in cancer therapy (6, 7). Ferroptosis is an iron-dependent, lipid peroxidation-driven form of programmed cell death. It is primarily characterized by glutathione (GSH) depletion, inhibition of glutathione peroxidase 4 (GPX4), and accumulation of reactive oxygen species (ROS). Morphologically, it manifests as compromised plasma membrane integrity, increased mitochondrial membrane density, and reduced mitochondrial cristae (8–10). Recent studies have demonstrated the antitumor potential of ferroptosis in various malignancies (11), including bladder cancer (12), gastric cancer (13), pancreatic cancer (14), and breast cancer (15). Notably, ALL cells exhibit a unique sensitivity to ferroptosis, offering a promising direction for the development of targeted therapies. This review provides an overview of the regulatory pathways governing ferroptosis, with a specific focus on its role in ALL, thereby laying a theoretical foundation for precision treatment and prognosis evaluation.

## 2 Overview of ferroptosis

**Figure 1:**
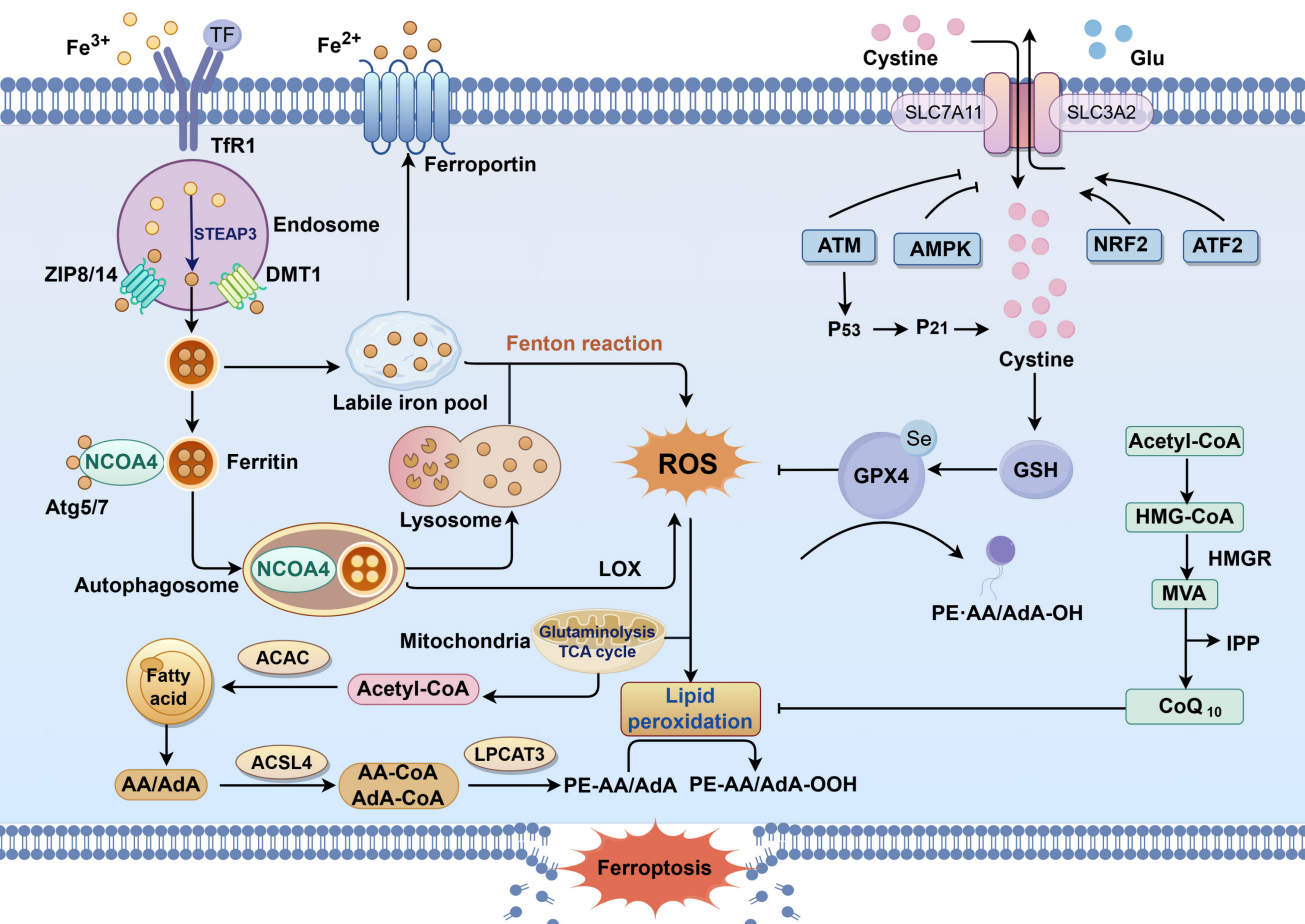
The mechanisms of ferroptosis.

Cells are the fundamental organizational units of life, and their proliferation, differentiation, and death are crucial for maintaining physiological homeostasis. Ferroptosis, an iron-dependent and lipid peroxidation-mediated form of cell death, involves a complex molecular regulatory network (16). The following sections elaborate on the specific regulatory mechanisms of ferroptosis from four perspectives: dysregulated iron metabolism, lipid metabolism pathways, antioxidant system imbalance, and autophagy regulation (**Figure 1**).

### 2.1 Dysregulated iron metabolism

Iron, an essential trace element, plays a vital role in numerous physiological processes, including oxygen transport, electron transfer, and enzymatic catalysis (17, 18). In the body, Fe^2+^ formed by intestinal absorption or red blood cell degradation can be oxidized to Fe^3+^ by ceruloplasmin. Fe^3+^ binds to transferrin (TF) and is transported into cells through the transferrin receptor (TFRC). It is reduced to divalent Fe^2+^ in the cell and stored in unstable iron pools and ferritin. Intracellular iron homeostasis relies on the balance between iron uptake, export, utilization, and storage. However, when iron metabolism is not ordered, a large amount of ferrous iron will undergo Fenton reaction with peroxide to produce Fe^3+^ and ROS (19). The production of these lipid peroxides and hydroxyl radicals will further promote the chain reaction of lipid free radicals and initiate or enhance ferroptosis (20). Therefore, regulating iron transport and absorption by using related inducers or inhibitors may be an effective way to regulate the level of ferroptosis in cells.

### 2.2 Lipid metabolism pathways

Uncontrolled lipid peroxidation is another key hallmark of ferroptosis. During this process, polyunsaturated fatty acids (PUFAs) such as arachidonic acid (AA) and adrenic acid (AdA) are esterified to phosphatidylethanolamine (PE) by acyl-CoA synthetase long-chain family member 4 (ACSL4) and lysophosphatidylcholine acyltransferase 3 (LPCAT3), forming AA-PE and AdA-PE conjugates (21, 22). Subsequently, these phosphatidylethanolamines are oxidized by 15-lipoxygenase (ALOX15) to form phospholipid hydroperoxides (PLOOH) (23). In the absence of reduction to the corresponding alcohols by GPX4 or other antioxidant pathways, this oxidation perpetuates, resulting in the accumulation of lipid peroxides. This chain reaction irreversibly damages the structure and function of cellular membranes, leading to ferroptotic cell death (24, 25).

### 2.3 Antioxidant pathways

Cellular redox balance is maintained by several antioxidant systems, including the GSH-GPX4, ferroptosis suppressor protein 1 (FSP1)- coenzyme Q10 (CoQ10), dihydroorotate dehydrogenase (DHODH)-CoQ10, and GTP cyclohydrolase 1 (GCH1)-tetrahydrobiopterin (BH4) pathways. Intracellular ROS levels directly influence lipid peroxidation, with GPX4 serving as the principal enzyme regulating ROS detoxification (26, 27). The cystine/glutamate antiporter System Xc^−^ facilitates cystine uptake, which is reduced to cysteine intracellularly and subsequently combined with glutamate and glycine to form GSH (28, 29). As the most abundant intracellular reducing agent, GSH provides reducing equivalents to GPX4, thereby mitigating oxidative stress and inhibiting ferroptosis (30). Thus, the synthesis of GSH, function of System Xc^−^, and activity of GPX4 collectively safeguard cells from ferroptosis under oxidative conditions.

In addition to the classical GPX4 pathway, the FSP1-CoQ10 pathway represents an independent antioxidant system. FSP1 reduces CoQ10 to ubiquinol using NADH, directly neutralizing lipid radicals and halting lipid peroxidation (31, 32). Similarly, DHODH contributes to ferroptosis resistance by catalyzing the mitochondrial reduction of CoQ10 (33). This process specifically occurs in the inner mitochondrial membrane and depends on the metabolic environment of the mitochondrial oxidative respiratory chain. Additionally, GCH1 protects against ferroptosis through its downstream product, BH4, which prevents oxidative degradation of phospholipids containing two PUFA chains (34, 35).

### 2.4 Autophagy regulates ferroptosis

Mitochondria are not only central to energy metabolism but also play critical roles in cancer development and the regulation of cell death. Voltage-dependent anion channels (VDACs), located on the outer mitochondrial membrane, serve as key transmembrane channels for the transport of ions and metabolites. The ferroptosis inducer erastin has been shown to act on VDACs, leading to mitochondrial dysfunction, altered membrane permeability, inhibition of glycolysis, and increased ROS release (36, 37).

Autophagy, a fundamental process for maintaining intracellular homeostasis, involves the degradation and recycling of cellular components (38). Some studies have suggested that disruption of iron homeostasis results in excessive accumulation of active iron within mitochondria, which triggers mitophagy. This, in turn, leads to the release of large amounts of divalent iron and its subsequent accumulation in the cytoplasm, thereby increasing cellular sensitivity to ferroptosis (39). Ferritinophagy, a selective form of autophagy, involves the binding of nuclear receptor coactivator 4 (NCOA4) to ferritin heavy chain 1 (FTH1), mediating its transport to lysosomes for degradation. This process releases free Fe^2+^ and regulates ROS production, thereby influencing the progression of ferroptosis (40, 41).

## 3 Candidate ferroptosis-related therapeutic targets in ALL

### 3.1 Iron metabolism-mediated regulation of ferroptosis in ALL

Beyond the disease itself, the tumor microenvironment (TME) plays a critical role in tumor progression and metastasis. The TME constitutes a multifaceted ecosystem composed of tumor cells, immune cells, stromal cells, and other components. Through molecular signals such as cytokines and their receptors, signal transduction networks, and various trace elements, tumor cells interact bidirectionally with their microenvironment, thereby reciprocally regulating each other’s biological behaviors (42). Iron is an essential nutrient that regulates numerous physiological processes within cells. Compared with non-malignant cells, cancer cells exhibit a heightened demand for iron to support their rapid growth and proliferation, making them more susceptible to ferroptosis. As a result, targeting iron metabolism has emerged as a promising strategy in the development of anti-cancer therapies. Iron chelators, which modulate intracellular iron concentrations and the activity of iron regulatory proteins, can suppress cancer cell invasiveness. For example, high-dose deferoxamine has been shown to disrupt iron homeostasis and induce apoptosis in breast cancer cells (43). The iron chelator deferasirox (44) has been reported to promote the death of multiple myeloma cells by inhibiting proline-rich tyrosine kinase 2. Benadiba et al. (45) reported that murine PTEN-deficient T-cell lymphoma and human T lymphoblastic leukemia/lymphoma cells exhibit characteristics of “iron addiction” in terms of survival and metabolism. Deferoxamine (DFO) chelates iron, thereby inhibiting iron-dependent antioxidant enzymes, leading to ROS accumulation and DNA damage in T-ALL cells. Moreover, combining DFO with chemotherapeutic agents such as L-asparaginase, dexamethasone, or doxorubicin, or with PARP inhibitors, has been shown to synergistically enhance anti-T-ALL effects, offering a novel strategy for combination therapies targeting iron metabolism. It has been reported that neutrophils exhibit sensitivity to ferroptosis induction. Disrupting the vicious cycle between Fer-CD4 T cells and neutrophils can effectively reverse chemotherapy resistance (46). Specifically, Fer-1 has the potential to prevent neutrophil cell death. As a result, when combined with the polo-like kinase inhibitor volasertib, it may generate additional therapeutic benefits (47). In addition, another research study indicates that the transcription factor MYB is highly expressed in various leukemia cells. MYB inhibits sorafenib-induced ferroptosis by upregulating FTH1, thereby endowing human leukemia cells with resistance to sorafenib. Consequently, the combination therapy of sorafenib with FTH1 or MYB inhibitors may hold the key to overcoming sorafenib resistance in patients (48).

### 3.2 Lipid metabolism-mediated regulation of ferroptosis in ALL

With the identification of PLOOHs as central mediators of ferroptosis, targeting lipid metabolism has become a promising strategy for inducing ferroptosis in ALL cells. Studies have shown that lipoxygenases (LOX) play a critical role in regulating ferroptosis in ALL. Experimental evidence indicates that LOX is involved in RSL3-induced lipid peroxidation and ROS bursts in ALL cells, triggering ferroptosis. Inhibition of ferroptosis in ALL cells by selective 12/15-LOX inhibitor baicalein and the pan-LOX inhibitor nordihydroguaiaretic acid has been observed, demonstrating their protective effects against RSL3-induced lipid peroxidation (49). These findings provide an experimental foundation for targeting lipid peroxidation in leukemia therapy.

Arsenic compounds are well-established as first-line treatments for acute promyelocytic leukemia (APL). Among these, the active component of the natural arsenic sulfide mineral realgar, tetraarsenic tetrasulfide (As_4_S_4_), has attracted attention for its potential application in ALL treatment. Bai et al. (50) demonstrated that As_4_S_4_ targets hexokinase-2 (HK2) in B-ALL cells to regulate the Warburg effect, inhibit glycolysis, induce ROS accumulation, activate the ROS/p53 signaling axis, and ultimately trigger ferroptosis. In APL, As_4_S_4_ has also been shown to induce cell death by modulating the Bcl-2/Bax/Cyt-C/AIF signaling pathway (51). Clinical research further supports the therapeutic advantages of As_4_S_4_, with evidence indicating superior efficacy and prognosis compared to arsenic trioxide (ATO), suggesting promising prospects for clinical application (52).

### 3.3 Antioxidant pathwathway-mediated regulation of ferroptosis in ALL

Targeting antioxidant pathways has become an important approach for regulating ferroptosis. The ferroptosis inducer erastin reduces intracellular GSH levels in T-ALL cells in a dose-dependent manner and downregulates the expression of SLC7A11 and GPX4. This process is positively regulated by the p38 MAPK signaling pathway and negatively regulated by the ERK1/2 pathway, with the dominant pro-ferroptotic effect mediated by p38 MAPK, ultimately leading to ferroptosis (53). Dihydroartemisinin (DHA), a derivative of the anti-malarial compound artemisinin (ART), has drawn significant interest due to its anti-cancer potential. DHA enhances the sensitivity of cancer cells to ferroptosis by depleting cysteine and inhibiting GPX4. In a study by Tang et al. (54), DHA was shown to induce ferroptosis in T-ALL cells by downregulating SLC7A11 and activating the ATF4-CHOP signaling pathway, which is associated with endoplasmic reticulum stress. This effect could be reversed by the ferroptosis inhibitor Ferrostatin-1. Another study indicated that AMP-activated protein kinase (AMPK), a central regulator of energy metabolism, when activated, can promote pro-apoptotic effects in ALL cells (55, 56). Recent findings have revealed that apigenin significantly downregulates GPX4 and SLC7A11 expression and upregulates the pro-ferroptotic protein ACSL4 by activating the AMPK signaling pathway (57).

Progress has also been made in the development of targeted drugs. The Niu team (58) developed a non-covalent peptide PROTAC targeting GPX4, referred to as Au-PGPD (peptide GPX4 PROTAC drug), based on the elevated GPX4 expression in ALL cells. This innovative strategy leverages the high expression of MDM2 in ALL cells to serve as an E3 ligase, selectively binding and degrading GPX4, thereby effectively inhibiting the proliferation of ALL cells. Furthermore, Ashoub et al. (59) utilized black cardamom extract to assist in the biosynthesis of zinc oxide nanoparticles (ZnO NPs), which were found to induce ferroptosis in Pre-B ALL cells (Nalm-6 and REH) by significantly reducing the mRNA-level expression of *SLC7A11* and *GPX4*, while upregulating the expression of *ACSL4* and *ALOX15*. Surprisingly, Au-PGPD and ZnO NPs exhibit no significant toxicity to normal cells, underscoring the selectivity and safety of the ferroptosis-targeted design. Nevertheless, a critical limitation of this study is the lack of *in vivo* experimental validation for the antitumor efficacy and biosafety of Au-PGPD and ZnO NPs. Evidence solely from cellular-level studies cannot adequately reflect their performance in complex living systems. Thus, subsequent research must urgently incorporate mouse models to specifically evaluate the *in vivo* pharmacokinetics, distribution, toxicity, and tumor-suppressive effects of these nanoparticles. Exploring their potential for combination therapies is also essential, as this will lay a crucial foundation for their future clinical translation.

### 3.4 Autophagy-mediated regulation of ferroptosis in ALL

Significant progress has been made in exploring the regulation of ferroptosis in ALL through autophagy. Pharmacological interventions targeting the autophagy pathway have been shown to markedly increase the sensitivity of ALL cells to ferroptosis, offering a novel therapeutic approach for ALL. In a study by Zhu’s research team (60), a combination of the autophagy activator rapamycin and erastin was used in an ALL xenograft mouse model. Silencing of the E3 ubiquitin ligase FBXW7, which specifically targets VDAC3, led to reduced VDAC3 ubiquitination and its increased protein stability. This enhancement significantly improved the responsiveness of ALL cells to erastin, thereby increasing their sensitivity to ferroptosis and prolonging the survival of the model mice. In the realm of natural medicine, the flavonoid lignan extract Hydnocarpin D (HD), derived from *Hydnocarpus anthelminticus*, has been found to induce autophagy by upregulating Beclin-1 and LC3-II expression. This process is accompanied by the downregulation of GPX4 protein and disruption of ROS homeostasis, ultimately inducing ferroptosis in T-ALL cells (61). Notably, HD also exerts anti-cancer effects in colon cancer models by inhibiting the Wnt/β-catenin signaling pathway (62). Additionally, poricoic acid A (PAA), a bioactive component of the traditional Chinese medicine Poria cocos, demonstrates potent anti-ALL effects. PAA induces autophagy in T-ALL cells through regulation of the AMPK/mTOR signaling axis and LC3 expression, while simultaneously inhibiting GPX4 activity. This leads to the accumulation of lipid peroxidation products and the induction of ferroptosis (63).

Analogous to normal hematopoietic stem cells, leukemia stem cells depend on a specific microenvironment to sustain self-renewal and survival (64). This bone marrow microenvironment, which consists of diverse cells and matrices, can be remodeled by leukemia cells to establish a niche that supports their proliferation, survival, and drug resistance (65). Previous research has demonstrated that the Notch signaling pathway can influence the development of T cells and B cells through interactions with the bone marrow and thymus microenvironments (66). The aberrant activation of the Notch signaling pathway has been closely associated with drug resistance in various cancers. Among these, *NOTCH1* mutations are frequently observed in T-ALL (67). Clinical evidence suggests that the mutational status of *NOTCH1* and *FBXW7* has prognostic value: patients with *NOTCH1/FBXW7* mutations tend to have favorable outcomes, whereas those without these mutations or with *RAS*/*PTEN* mutations exhibit poorer prognoses. This finding suggests that the Notch pathway can be used as a biomarker for molecular stratification of T-ALL (68–71). Targeted therapeutic strategies against the Notch pathway have also shown promise. γ-Secretase inhibitors (GSIs), which block the proteolytic activation of Notch receptors, have demonstrated efficacy in targeting leukemia-initiating cells *in vitro* and in clinical trials (72–74). However, the effectiveness of GSI monotherapy is limited, spurring interest in combination therapies. For example, Cullion et al. (73) demonstrated that combining GSI with rapamycin significantly enhanced the growth-inhibitory effect on T-ALL cell lines and improved survival in a mouse transplant model. Subsequent mechanistic studies by Herranz et al. (75) revealed that autophagy induction could synergize with anti-Notch1 therapy, providing a new avenue for treating refractory ALL. Current research is increasingly focused on the interplay between the Notch pathway, autophagy, and ferroptosis, which may drive the clinical implementation of precision medicine strategies targeting Notch.

### 3.5 Genes that regulate ferroptosis in ALL

Cancer cells may exploit genetic mechanisms to counteract metabolic and oxidative stress, such as the upregulation of SLC7A11 or the antioxidant transcription factor NRF2, indicating that the genetic background of a tumor plays a decisive role in ferroptosis regulation. Circular RNAs (circRNAs), a class of non-coding RNAs widely expressed in eukaryotic cells, have been shown to exert regulatory control in various malignancies. Studies have revealed that circ_0000745 is upregulated in ALL and activates the ERK signaling pathway to promote ALL cell viability (76). Further investigation demonstrated that circ_0000745 acts as a molecular sponge for miR-494-3p, thereby inducing the expression of the downstream target gene NET1 and suppressing ferroptosis in ALL cells. Conversely, the knockdown of circ_0000745 disrupts cell cycle progression and glycolysis, ultimately promoting ferroptosis (77). These findings highlight the regulatory role of circRNAs in ferroptosis within the context of ALL.

PAQR3, a known tumor suppressor, is characterized by low expression across multiple malignancies, including ALL, osteosarcoma, glioma, breast cancer, and non-small cell lung cancer (78). In ALL, PAQR3 modulates the stability of NRF2, a key regulator of oxidative stress, via the ubiquitination pathway, thereby promoting ferroptosis in ALL cells (79). Furthermore, In ALL cell lines, the ferroptosis suppressor protein 1 (FSP1) is inhibited in expression due to high promoter methylation, leading to a greater reliance of cancer cells on the glutathione (GSH)-dependent anti-ferroptosis pathway. This makes ALL cells sensitive to compounds targeting the GSH pathway, while overexpression of *FSP1* can enhance resistance and promote tumor growth, revealing the metabolic vulnerability and therapeutic potential of ALL (80).

Collectively, these studies not only underscore the critical role of ferroptosis in ALL pathophysiology but also provide a theoretical and practical foundation for developing effective, low-toxicity targeted therapies (**Table 1**).

**Table 1:** Experimental studies of drugs or monomers for ferroptosis treatment of ALL.

Active ingredients	Model	Mechanism(s) of action	References
DFO	Murine PTEN-deficient T lymphoma and human T lymphoblastic leukemia/lymphoma	Regulating iron concentrations	(45)
LOX	Human T-ALL cell lines (Jurkat and Molt-4)	Lipoxygenase LOX regulates RSL3-induced lipid peroxidation	(49)
As_4_S_4_	Human B-ALL cell lines (Nalm-6 and RS4; 11)	HK2/ROS/p53	(50)
erastin	Human T-ALL cell lines (Molt-4)	p38 MAPK and ERK1/2 pathways	(53)
DHA	Human T-ALL cell lines (Jurkat and Molt-4)	Inhibition of SLC7A11 expression and activation of the ATF4-CHOP pathway	(54)
Apigenin	Human T-ALL cell lines (Jurkat) and B-ALL cell lines (SUP-B15)	Activation of AMPK	(57)
Au-PGPD	The GSE13159 dataset	GPX4 binding and selective degradation	(58)
ZnO NPs	Human B-ALL cell lines (Nalm-6 and REH)	Decrease GPX4 expression	(59)
Rapamycin combined with erastin	Human T-ALL cell lines (Jurkat and CCRF-CEM)	FBXW7/VDAC3	(60)
HD	Human T-ALL cell lines (Jurkat and Molt-4)	Autophagy depends on ferroptosis	(61)
PAA	Human T-ALL cell lines (Jurkat, Molt-3, ALL-SIL and RPMI-8402)	AMPK/mTOR signaling axis and LC3	(63)

## 4 Molecular markers of ferroptosis in ALL

In recent years, increasing attention has been paid to the prognostic stratification of ALL based on molecular markers. Tian et al. (81) selected differentially expressed genes (DEGs) from the GSE46170 database, then cross-compared them with ferroptosis-related genes (FRGs) in the FerrDb database to screen out the iron death-related DEGs. Through PPI network analysis, five ferroptosis hub genes related to ALL were identified: *LCN2, LTF, HP, SLC40A1*, and *TFRC*. Among these, LCN2 is highly expressed in T-ALL, and its silencing has been shown to significantly increase the sensitivity of T-ALL cells to ferroptosis. Lalonde et al. (82) conducted a genome-wide CRISPR screen in B-ALL and identified ferroptosis-related genes, including *PAX5, RUNX1, TCF3, EBF1, CD79A, CD79B*, and *PIK3CD*. Similarly, Hong et al. (83) analyzed clinical data and RNA-seq results from 80 Ph-negative B-ALL patients and identified eight FRGs: *ALOX15, ATP5G3, CARS, CDKN1A, LPCAT3, SAT1, SLC1A5*, and *TFRC*. These studies affirm the prognostic significance of FRGs in ALL, independent of traditional clinical parameters. Collectively, these hub genes serve as valuable targets for the development of precision therapies targeting ferroptosis and hold promise as reliable prognostic biomarkers for ALL.

## 5 Summary

Ferroptosis, as a relatively novel form of programmed cell death, presents a promising direction for improving treatment and prognostic evaluation in ALL. The existing evidence indicates that targeting the iron metabolism pathway or regulating the antioxidant system can effectively trigger ferroptosis in leukemia cells, providing new molecular targets and strategies for the treatment of ALL (especially refractory/relapsed cases). Basic research has fully demonstrated the dual value of inducing ferroptosis in the treatment of ALL: it can be used as an independent anti-tumor agent and can also be combined with traditional chemotherapy drugs to significantly enhance the responsiveness of leukemia cells. The combination of ferroptosis induction with traditional anti-tumor treatment methods such as chemotherapy and immunotherapy may have more clinical translational significance. In recent years, new platforms for precise delivery, such as nanobiotechnology and antibody-drug conjugates, have emerged continuously. These new approaches, such as iron-based nanomaterials and functionalized polymer nanocarriers, are expected to effectively enhance the efficiency of drug delivery, improve the anti-tumor effect by enhancing targeting and bioavailability, and significantly reduce systemic toxicity, demonstrating great potential in clinical applications.

Despite mounting evidence supporting its critical role in ALL, the intricate and interconnected signaling pathways involved remain incompletely understood. To date, research in this field has primarily relied on *in vitro* and animal models. Therefore, it is imperative to expand clinical validation efforts, accelerate the translation of basic findings into clinical practice, and pursue more experimental studies focusing on ferroptosis-targeted drug interventions for ALL prevention and treatment. Additionally, the potential of combining ferroptosis inducers with conventional chemotherapeutics or agents with different mechanisms warrants thorough investigation. Such combination strategies could offer more effective treatment regimens. As research into ferroptosis in ALL progresses, the development and clinical evaluation of targeted therapeutics is expected to drive significant breakthroughs in precision treatment and prognosis management for ALL patients. It is expected that in-depth exploration of the related mechanisms and clinical translation in the future will bring new hope to more patients.
